# Leeds-Genoa Non-Union Index: a clinical tool for asessing the need for early intervention after long bone fracture fixation

**DOI:** 10.1007/s00264-019-04376-0

**Published:** 2019-08-22

**Authors:** Emmanuele Santolini, Robert M. West, Peter V. Giannoudis

**Affiliations:** 1 Academic Unit of Trauma and Orthopaedics, University of Genoa, Ospedale Policlinico San Martino, Largo R. Benzi 10, 16132 Genoa, Italy; 2 Academic Department of Trauma and Orthopaedics, School of Medicine, University of Leeds, Leeds General Infirmary, Clarendon wing, Level D, LS13EX, Leeds, West Yorkshire UK; 3 Leeds Institute of Health Sciences, University of Leeds, 101 Clarendon Road, Leeds, UK; 4 NIHR Leeds Biomedical Research Center, Chapel Allerton Hospital, Leeds, UK

**Keywords:** Non-union, Prediction index, Early intervention, Femur, Tibia

## Abstract

**Aim of the study:**

The aim of this case–control study was to develop a clinical decision rule to support assessment of the risk of long-bone non-union and plan for appropriate early intervention.

**Methods:**

Two hundred patients (100 cases and 100 controls) were recruited. Risk factors identified to contribute to the development of non-union were recorded and analysed with a multivariable logistic regression model. Tabulation of the outcome (non-union/union) against each risk factor in turn (univariable analysis) was carried out. Odds ratios and confidence intervals were derived using Wald’s method. A receiver–operator curve was calculated and the area under the curve was computed. Having established the eight most important risk factors, a non-union risk index was developed as the count of the risk factors present in each patient.

**Results:**

The five risk factors for non-union with greater effect size were post-surgical fracture gap > 4 mm (odds ratio (OR) = 11.97 95% CI (4.27, 33.53)), infection superficial/deep (OR 10.16 (2.44, 42.36)), not optimum mechanical stability (OR 10.06 (3.75, 26.97)), displacement > 75% of shaft width (OR 6.81 (2.21, 20.95)), and site of fracture—tibia (OR 4.33 (1.32, 14.14)). The ROC curve for the non-union index was 0.924, sensitivity 91%, specificity 77%.

**Conclusions:**

The non-union index derived from counting risk factors predicts union for 0–4 risk factors and non-union for 5–8 risk factors. It can be readily applied and can guide clinicians about the risk of development of long-bone non-union. It can become a powerful aid for assessing fracture fixation outcome and to support early intervention.

## Introduction

Long-bone non-union continues to be one of the most common post-fracture fixation complication with an incidence reported to range between 5 and 10% [1–3]. Its impact in terms of resource allocation and utilization can be substantial [4–6]. Currently, non-union according to the Food and Drugs Administration (FDA) agency is defined as a fracture being over nine months old with no signs of progression toward healing for three months [7]. However, assessing the risk of developing non-union early, within 12 weeks after fracture fixation, could be helpful for planning to intervene early and could lead not only to faster patient recovery but also to a reduction of utilization of clinical resources [8]. Previous studies have examined the value of biomarkers for predicting non-union, but up to now, whilst this work is promising, this approach has not been effective [9]. Consequently, the availability of a clinical decision rule, which could provide the orthopaedic surgeons with the extent of the risk of developing non-union, would assist them to formulate an early re-intervention plan, contrary to the usual established ‘wait and see policy’.

The aim of this case–control study was to explore the development and effectiveness of a clinical decision rule, which would provide valuable feedback in terms of the risk of developing non-union within 12 weeks after initial fixation of a fracture of the femoral or tibial shaft. This clinical decision rule would define a high-risk group for developing an impaired fracture healing response.

## Materials and methods

Initially, we undertook a comprehensive evaluation of the literature in order to identify risk factors contributing to non-union of the femur and tibia [10]. Overall, ten risk factors with large effect sizes and good evidence were used to build a scoring model. The relative effects on the fracture of these factors were assessed by the use of a case–control approach.

Consequently, a retrospective case–control study was conducted at two level I trauma centres: Leeds General Infirmary, Leeds, UK, and Ospedale Policlinico San Martino, Genoa, Italy. Institutional board review approval was obtained from both institutions (LTH - 911 and OPSM-140REG2016) for the study. Using the orthopaedic trauma registries of the two departments, all the femoral and tibial non-unions treated between June 2009 and September 2016 were identified retrospectively. From the same period of time, randomly selected patients with femoral and tibial fractures that progressed uneventfully to union formed the control group of the study.

### Study participants

Inclusion criteria required patients to be adults (≥ 18 years of age) having sustained a tibial and/or femoral shaft non-union following surgical stabilization. We considered as femoral shaft the segment between the lower edge of the lesser trochanter and the metaphyseal zone at least 5 cm above the femoral condyles. The tibial shaft was assigned as the segment between below the tibial tubercle and at least 3 cm above the tibial plafond distally. Non-unions that occurred in other segments of the femur and tibia and other long bones were excluded as well as fractures not surgically treated, pathological fractures due to tumours, open fractures associated with significant bone loss (defects), periprosthetic fractures, and patients with such conditions as osteogenesis imperfecta and Paget disease. In addition, segmental fractures were excluded as there are inherent difficulties in a standardized evaluation due to the variable length and location of the intermediate segment.

As surgical treatment, we consider only the three standard definitive methods of treatment including intramedullary nail (IM), plate, and circular external fixator. Patients managed definitively with axial external fixators were also excluded.

### Collection of data

We developed a proforma for the accurate and consistent documentation of data. Both radiographs and clinical records of each patient were carefully evaluated, and all risk factors contributing to the development of non-union were documented. Radiographs were assessed for the state of fixation and state of mechanical stability according to the type of implant used. The fracture pattern and the location of non-union were also recorded. In addition, the initial degree of fracture displacement was documented as well as the degree of any post-fixation surgical fracture gap if present. From the clinical records, such details were extracted as the initial state of the soft tissue envelope at presentation, method of reduction during surgery, patient smoking habit, development of infection, diabetes, peripheral vascular disease, and other relevant medical details. As our institutions have stopped using non-steroidal anti-inflammatory drugs (NSAIDs) post-operatively for analgesia since 2007 due to their negative effects on bone healing, this parameter was not considered. Patients with incomplete documentation, either for radiographs or for medical records, were excluded. Patients were not matched on either demographics or clinical characteristics, but both cases and controls were selected from the same time period (2009–2016).

### Study size

Up to ten risk factors were considered based on the strongest evidence from the literature review [10]. To robustly assess ten variables in a logistic regression, it is necessary to have 100 events (10 times the number of variables [11]), and consequently, 100 cases and 100 controls were recruited to the study.

#### Variables—definitions and measurements

##### Non-union

Non-union was defined as a fracture not able to heal without any further intervention within nine months of the initial surgical treatment, evidenced by no progression from three consecutive months of serial radiographs [7].

#### Ten risk factors for non-union

i. Displacement. Initial deformity of the bone after a fracture, expressed as the degree of dislocation of each bone fragment over another one. We considered displacement as minimal or moderate when smaller than 75% of the shaft width and marked when greater than 75% of the shaft width [12]. The degree of displacement was evaluated on the first radiograph taken, at the highest point of the deformity regardless of the projection (anterior–posterior (ap) or lateral). In cases where due to application of a temporarily external fixator the displacement of the fragments increased compared to the original position at presentation, then this later degree of displacement was considered.
ii. Anatomical location of fracture. Fracture that occurred in the tibial shaft rather than in the femoral shaft was considered as a location with a greater risk of non-union [2].
iii. Fracture location according to the vascularization of the bone. The femoral and tibial shafts were divided into sections of thirds according to the degree of vascularization previously described [13]. Accordingly, for the tibia, the upper third has a rich vascularization, the middle third has a moderate vascularization, and the lower third has a deprived vascularization; For the femur, the upper third has a moderate vascularization, the middle third a rich vascularization, and the lower third a poor vascularization. Particular emphasis was given in fractures and subsequent non-unions which occurred in areas of low vascularization.
iv. Infection. Infection being either superficial or deep was defined according to the Centers for Disease Control criteria [14]. The presence of superficial or deep infection was given the same gravitas, as many superficial infections not infrequently represent and/or become deep infections.
v. Method of reduction. The type of reduction being closed or opened was given a different degree of importance. Opened reduction rather than closed is related with a higher incidence of non-union [15]. When the surgeon used an aid in order to achieve the best possible reduction utilizing a minimal opening at the fracture site (i.e. by using a pointed reduction forceps or other surgical instruments), then, in these cases, the method of reduction was considered as closed. For Gustilo grade II and III [16] open fractures, the method of reduction was considered as opened.
vi. Mechanical stability. It relates to the type of surgical technique and implant used to stabilize the fracture (intramedullary nailing, plating, and external fixators) [17]. We standardized the way to determine the degree of mechanical stability of each fracture fixation technique in order to be able to compare them. Table 1 (A, B, C) specifies the standardization process in order to assess the degree of fracture stability in each patient on the basis of the type of surgery performed. Two levels of stability were assigned for each surgical technique, either optimum or not optimum stability. Optimum stability was defined if all the three rules defined from the outset were respected; otherwise, the degree of stability was defined as not optimum. The degree of mechanical stability present was assessed by the evaluation of post-operative radiographs [18–28].
vii. Presence of post-fixation fracture gap. It was defined as the existing residual gap between the bony fragments after surgical fixation [29]. Evaluation was based on the post-operative radiographs by measuring the gap size at the point of the greatest gap regardless of the projection (AP or lateral). Patients were stratified based on whether their gap was less than or equal to 4 mm or greater than 4 mm.
viii. Smoking habit. Smoking has been associated with a higher incidence of non-union [30]. Smoking was assessed from the medical records and where necessary by interviewing the patients in the clinics. We considered as smokers patients smoking more than five cigarettes per day [31].
ix. Soft tissue damage. Both closed and open fractures were considered for this variable. Regarding closed fractures, soft tissues were graded as damaged in case of internal degloving, a condition that occurs in association with fractures with a degree of displacement greater than 100% of the shaft width or in case of type C complex fractures. Internal degloving in closed fractures was evaluated on the first radiograph taken. On the other hand, opened fractures were graded as having inherent soft tissue damage from the outset irrespectively of the grade of injury [32].
x. Type of fracture. According to the AO classification [33] of the diaphyseal fractures, we contemplated three main fracture types: simple (type A), wedge (type B), and complex (type C). Wedge or complex types of fracture present a higher risk of non-union than simple fracture types. Type of fracture was evaluated on the first radiograph taken.

**Table 1:** Diagram for evaluation of mechanical stability

A. Diagram for plating techniques	
Conventional plate [22, 23, 26, 27]	
Parameters	1 - ≥ 3 screws for each main fragment
	2 - use of lag screw
	3 - good quality of the bone
Optimum	1 + 2 + 3
Not optimum	Not all of them
Locking plate [18, 23, 26, 27]	
Parameters	1 - ≥ 3 screws for each main fragment
	2 - length of plate–plate span ratio^a^: > 2–3 comminuted #, > 8–10 simple #
	3 - distance between the plate and the bone < 5 mm
Optimum	1 + 2 + 3
Not optimum	Not all of them
B. Diagram for nailing techniques	
Nail [19, 24, 28]	
Parameters	1 - ≥ 2 proximal and distal interlocking screws
	2 - reamed nail
	3 - largest possible nail diameter obtaining nail-cortical contact
Optimum	1 + 2 + 3
Not optimum	Not all of them
C. Diagram for circular external fixator techniques	
Ex fix—circular [20, 21, 25]	
Parameters	1 - number of rings: ≥ 2 for each fragment
	2 - appearance of the wires as straight
	3 - proper length of the frame: from proximal to distal physis line
Optimum	1 + 2 + 3
Not optimum	Not all of them

***#*** Fracture

^a^Ratio between plate length and the overall fracture length. It must be higher than 2–3 in comminuted fractures and 8–10 in simple fractures

### Bias

Cases were selected from consecutive records of the registries. Controls were randomly selected from the same time period. In this way, selection bias was minimized. There was no concern regarding recall bias since all factors were collected from patient notes/records. Records were only included if there was complete data available, so it is possible that the study patients were not representative of all patients in the registries. The requirement for full records was the same for both the study and the control groups respectively.

### Statistical analyses

The risk factors for non-union as previously stated were established from the clinical literature. The task here was to determine the relative effect sizes when all were considered concurrently. This was achieved with a multivariable logistic regression.

Tabulation of the outcome (non-union/union) against each risk factor in turn (univariable analysis) was carried out. Adjusted effect sizes were determined using a multivariable logistic regression with all ten factors concurrently. Adjusted odds ratios and confidence intervals were derived using Wald’s method.

Having established the most important risk factors, a non-union risk index was proposed as the count of the risk factors present in each patient. This follows the approach taken by Clegg et al. [34] in the development and validation of a frailty index and is supported in the statistical literature by, for example, Dawes [35]. The aim was to develop a robust index which can be quickly calculated in routine clinical care.

To validate the non-union risk index, the Hosmer–Lemeshow test was applied. The performance of the usual logistic regression weighting was also assessed to demonstrate that the index loses little discriminatory power.

All statistical analysis was undertaken with R statistical software version 3.3.2 [36], and the Hosmer–Lemeshow test used the R library 0.2–6 [37].

The resulting clinical decision support rule was call the Leeds–Genoa Non-Union Index: LEG-NUI.

## Results

**Fig. 1:**
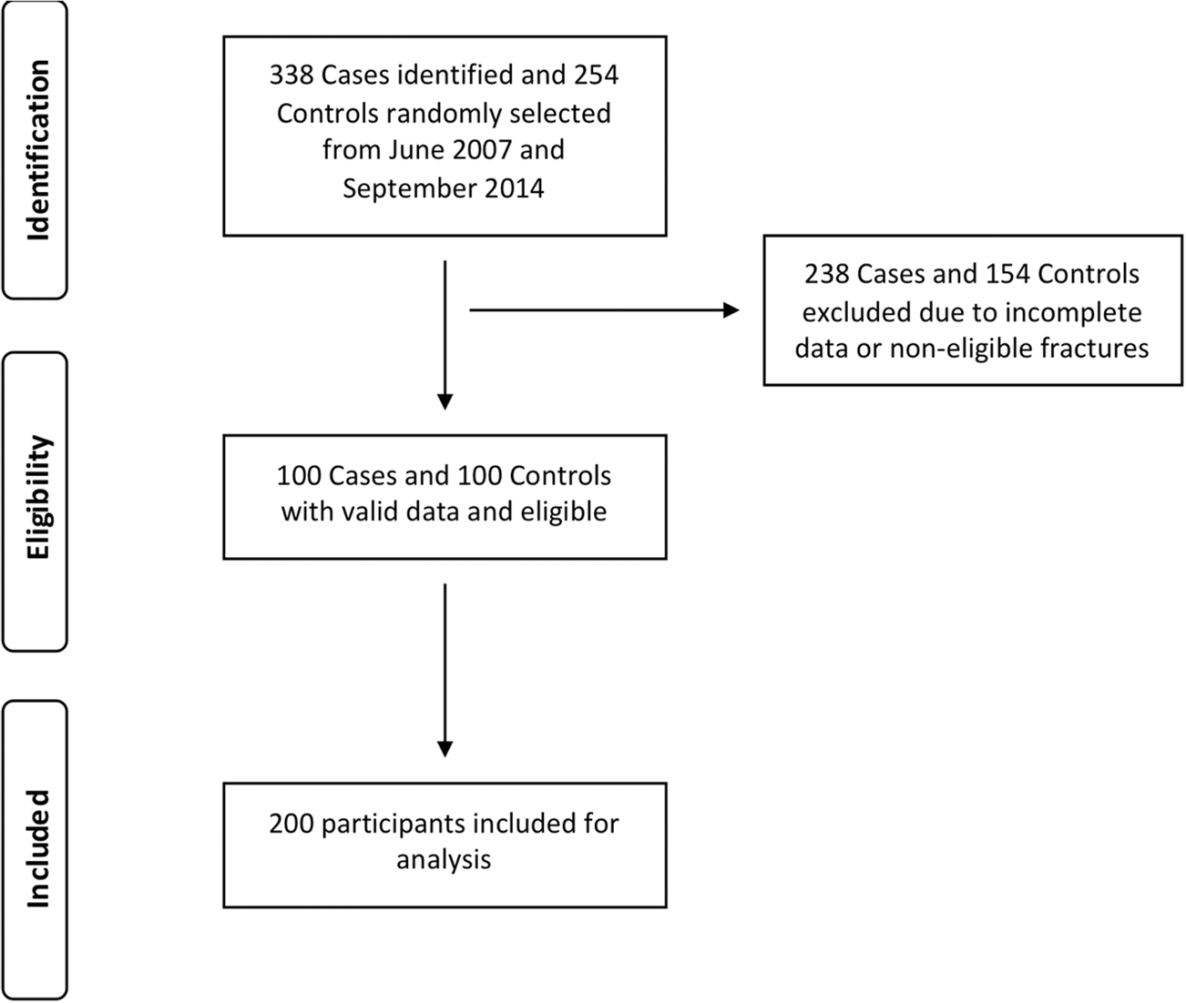
Participant flow diagram

A total of 338 study cases and 254 randomly selected controls were identified from the hospital registries. Of these, 238 cases and 154 controls were excluded due to non-eligibility based on the inclusion–exclusion criteria or to having incomplete data. Consequently, 100 eligible cases and 100 eligible controls were identified. Thus, in total, 200 participants were included for statistical analysis and formed the basis of this study (Fig. 1).

Demographic and risk factors for the study participants are tabulated in Table 2, complete with a Pearson’s chi-square test to permit easier comparison between the union and non-union groups.

**Table 2:** Tabulation of demographic and risk factors by non-union outcome

		Non-union	Union	*P* value chi-sq. test or *t* test
Demographic factors				
Number of patients		100	100	
Sex	Female	34	41	
	Male	66	59	0.381
Age mean (SD)		46.6 (18.4)	44.7 (20.7)	0.475
Risk factors				
Post-surgical fracture gap	≤ 4 mm	16	65	
	> 4 mm	84	35	< 0.001*
Infection	None	75	95	
	Superficial or deep	25	5	< 0.001*
Mechanical stability	Optimum	34	77	
	Not optimum	66	23	< 0.001*
Displacement	< 75% shaft width	26	66	
	> 75% of shaft width	74	34	< 0.001*
Site of fracture	Femur	55	42	
	Tibia	45	58	0.090
Soft tissue damage	Closed fracture with no degloving	10	59	
	Closed fracture with degloving or open fracture of any grade	90	41	< 0.001*
Method of reduction	Closed	40	73	
	Opened	60	27	< 0.001*
Type of fracture	Simple	15	41	
	Wedge or complex	85	59	< 0.001*
Smoking habit	Non-smoker	56	65	
	Current smoker	44	35	0.247
Fracture location according to areas of vascularization of the bone	Area of medium/high vascularization	42	42	
	Area of low vascularization	58	58	1.000

* Statistically significant

The results from fitting a logistic regression with the ten risk factors are given in Table 3, which provides both adjusted and unadjusted odds ratios and 95% confidence intervals. No demographic factors were included in the model since they did not substantially alter the model.

**Table 3:** Unadjusted and adjusted odds ratios for 10 risk factors associated with non-union

Risk factors	Unadjusted OR (95% CI)	Adjusted OR (95% CI)
Post-surgical fracture gap > 4 mm	9.75 (4.97, 19.14)	11.54 (4.12, 32.33)
Infection superficial or deep	6.33 (2.31, 17.33)	9.98 (2.40, 41.46)
Not optimum mechanical stability	6.50 (3.49, 12.12)	9.60 (3.57, 25.87)
Displacement > 75% of shaft width	5.53 (3.01, 10.16)	6.69 (2.19, 20.42)
Site of fracture—tibia	0.59 (0.34, 1.04)	4.08 (1.24, 13.39)
Closed fracture with internal degloving or open fracture	12.95 (6.03, 27.84)	3.99 (1.24, 12.81)
Open method of reduction	4.06 (2.24, 7.36)	3.90 (1.46, 10.44)
Wedge or comminuted type of fracture	3.94 (2.00, 7.76)	3.20 (1.03, 9.99)
Patient current smoker	1.46 (0.83, 7.76)	1.39 (0.53, 3.63)
Fracture location in the area of low vascularization	1.00 (0.57, 1.75)	1.31 (0.51, 3.35)

A receiver–operator curve was calculated and the area under the curve was 0.938. At the optimal cut point, the sensitivity was 91% and the specificity 82%. It is clear, however, that the evidence from smoking status and vascularization area was limited. So, these factors were dropped and the fitted model became that as expressed in Table 4.

**Table 4:** Unadjusted and adjusted odds ratios for 8 risk factors associated with non-union

Risk factors	Unadjusted OR (95% CI)	Adjusted OR (95% CI)
Post-surgical fracture gap > 4 mm	9.75 (4.97, 19.14)	11.97 (4.27, 33.53)
Infection superficial or deep	6.33 (2.31, 17.33)	10.16 (2.44, 42.36)
Not optimum mechanical stability	6.50 (3.49, 12.12)	10.06 (3.75, 26.97)
Displacement > 75% of shaft width	5.53 (3.01, 10.16)	6.81 (2.21, 20.95)
Site of fracture—tibia	0.59 (0.34, 1.04)	4.33 (1.32, 14.14)
Closed fracture with internal degloving or open fracture	12.95 (6.03, 27.84)	3.74 (1.19, 11.80)
Open method of reduction	4.06 (2.24, 7.36)	3.88 (1.47, 10.27)
Wedge or comminuted type of fracture	3.94 (2.00, 7.76)	3.48 (1.15, 10.58)

**Fig. 2:**
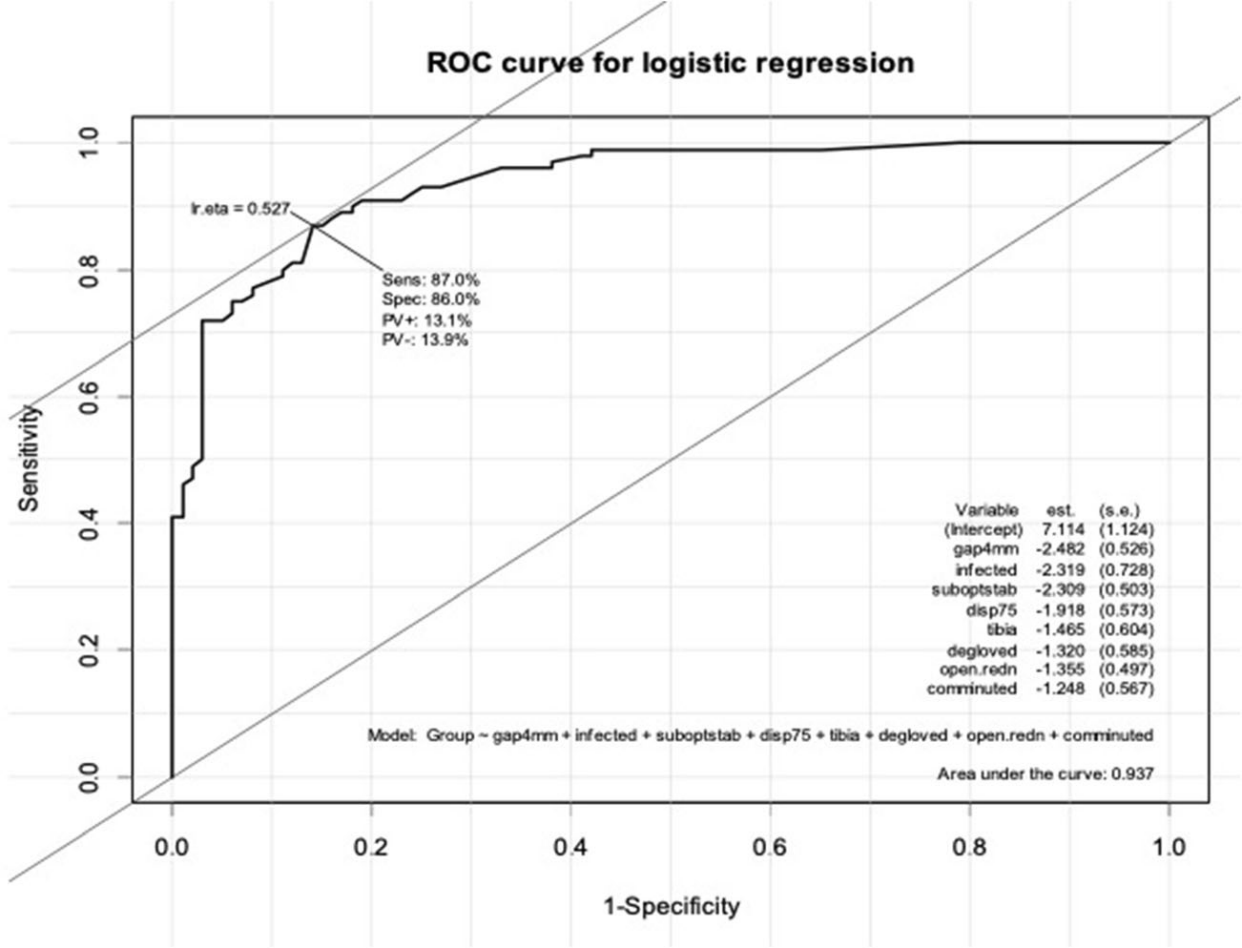
ROC curve for rule derived from 8-factor logistic regression

Note that the area under the curve was reduced only very slightly to 0.937, and at the optimal cut point, the sensitivity was 87% and specificity 86%. These eight risk factors all had large effect sizes and good statistical significance. The ROC curve for the logistic regression on eight factors is shown in Fig. 2. The Hosmer–Lemeshow test with ten groups yielded a chi-square value of 4.37 on eight degrees of freedom, *p* = 0.822, confirming a good fit.

**Fig. 3:**
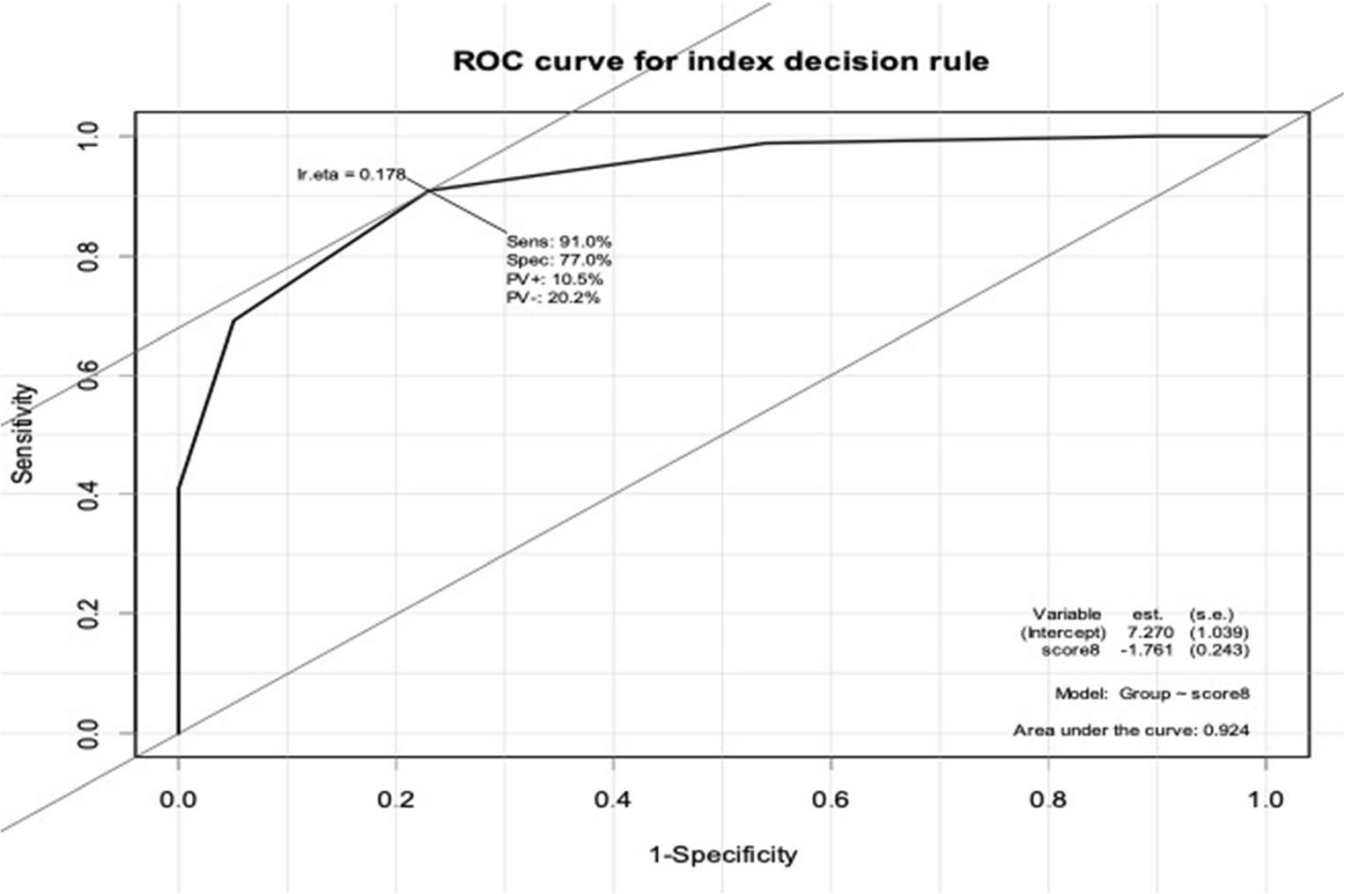
ROC curve for the non-union index derived the sum of the risk factors

The non-union index, which was the sum of the risk factors for each patient, performed almost as well. The ROC curve is shown in Fig. 3, area under the curve 0.924, sensitivity 91%, specificity 77%—see Tables 5 and 6. For the non-union index, the Hosmer–Lemeshow test with ten groups yielded chi-square = 1.26, *p* = 0.996.

**Table 5:** Classification table for non-union index

Decision rule based on the non-union index	Non-union	Union
Predicted non-union	77	9
Predicted union	23	91

**Table 6:** Non-union index by fracture outcome

Non-union index	Non-union	Union
1	0	18
2	0	23
3	5	28
4	18	22
5	31	8
6	36	1
7	9	0
8	1	0

**Fig. 4:**
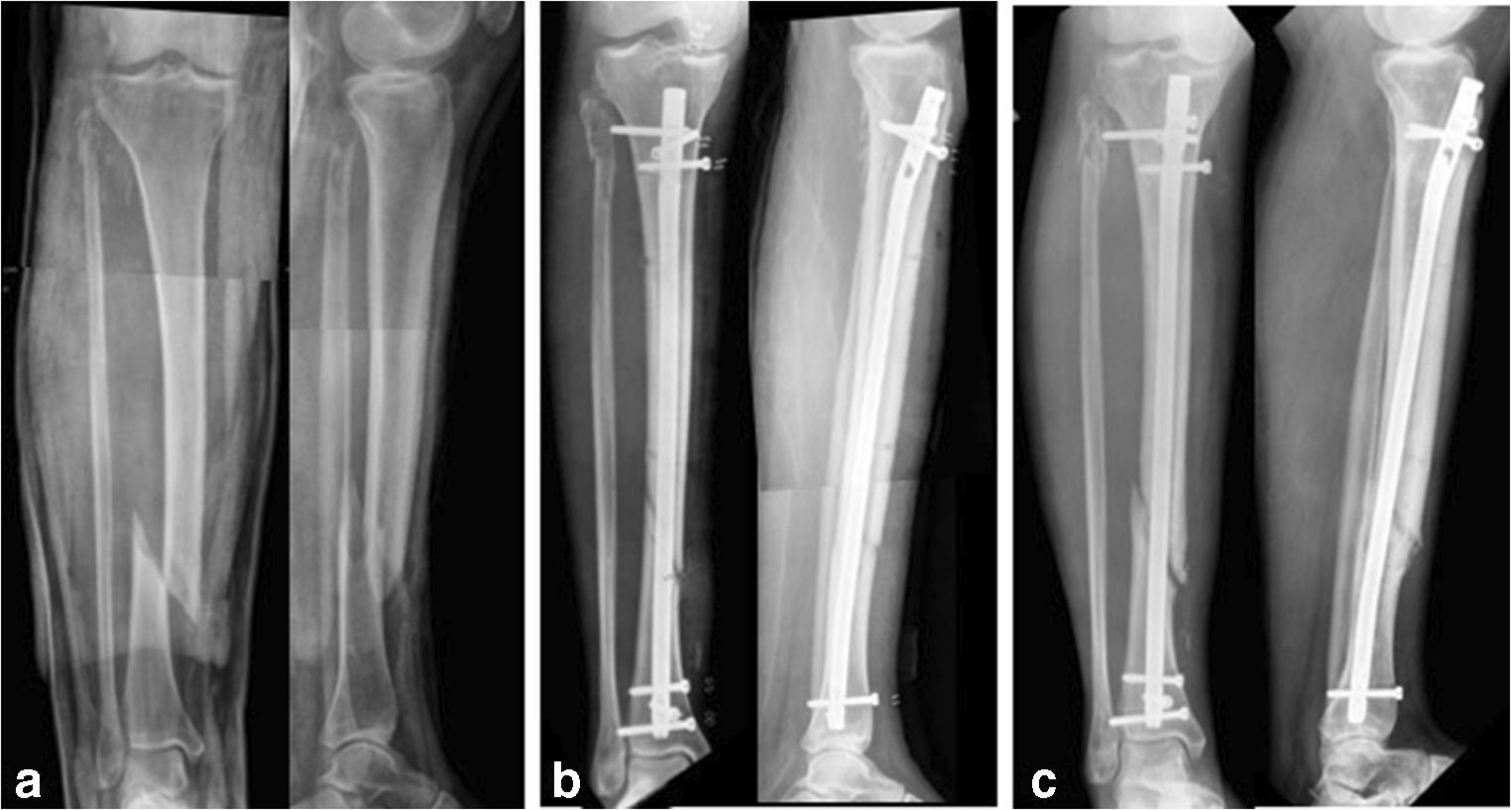
Patient 1: **a** Preoperative x-rays of distal tibial fracture. **b** Post-operative x-rays with intramedullary nail fixation. **c** 8-month follow-up films showing distal tibial non-union. The score calculated was 7 points (see Table 7)

**Fig. 5:**
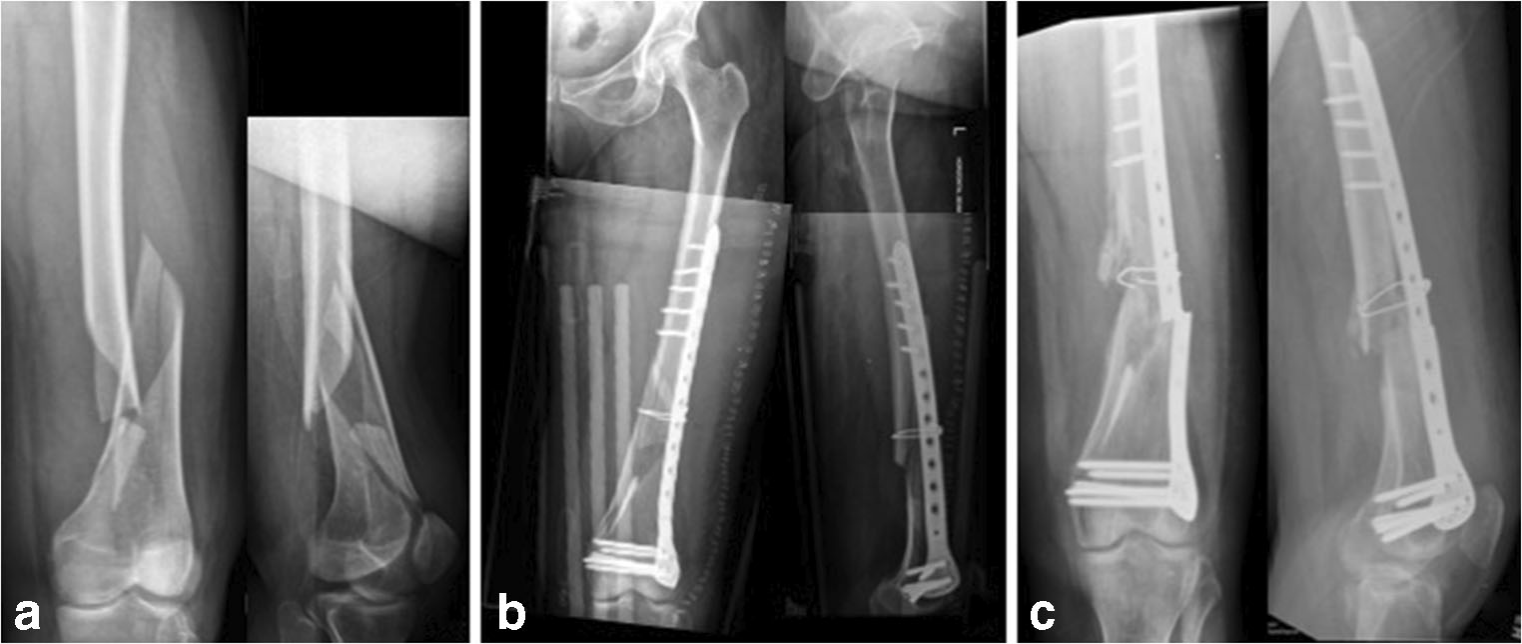
Patient 2: **a** Preoperative x-rays of distal third femoral diaphyseal fracture. **b** Post-operative x-rays with a locking plate with screws and a cerclage wire. **c** 9-month follow-up x-rays showing distal femoral non-union with implant breakage. The score calculated was 5 points (see Table 8)

**Fig. 6:**
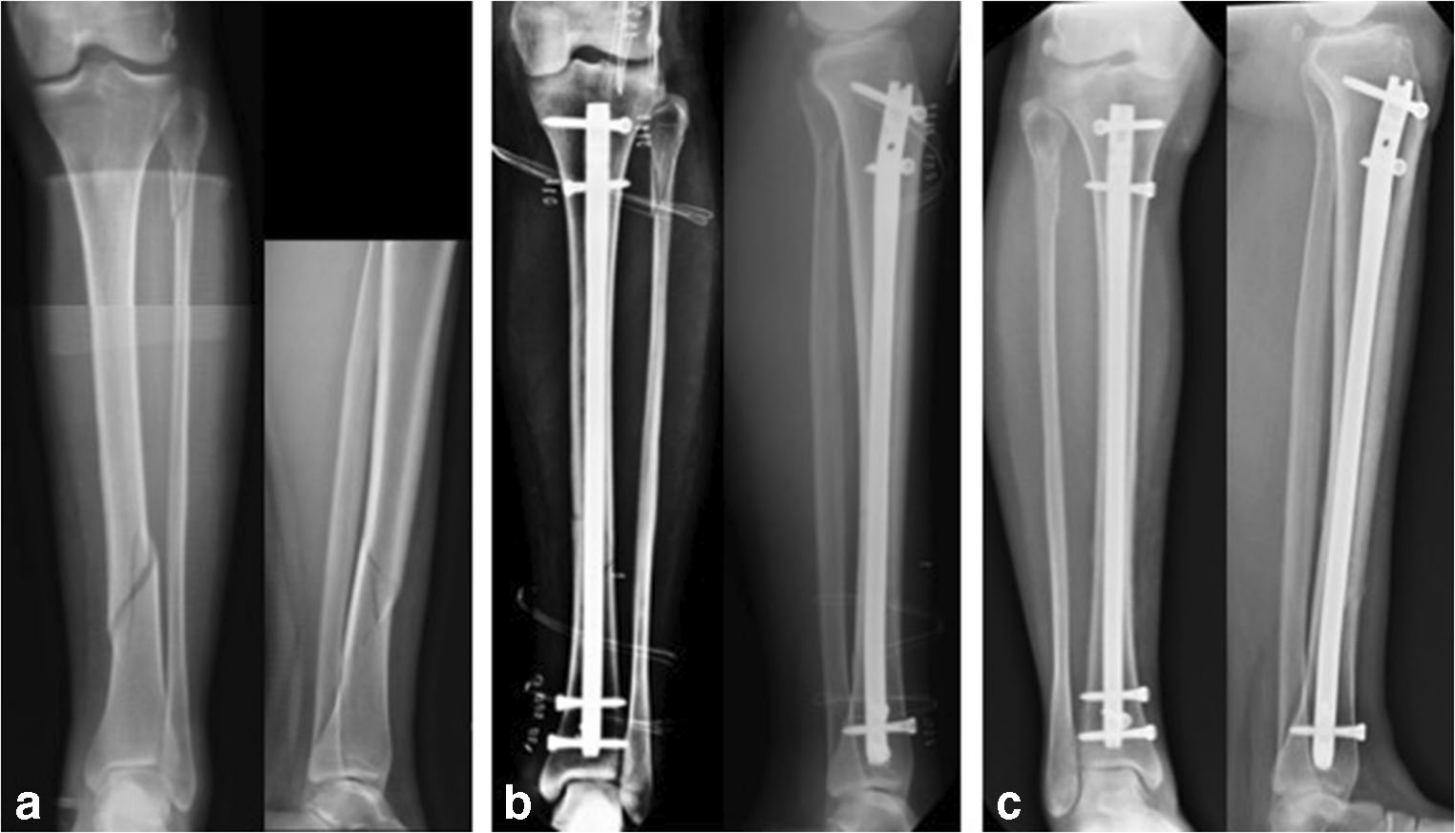
Patient 3: **a** Preoperative x-rays of distal third tibial diaphyseal fracture. **b** Post-operative x-rays with an intramedullary nail. **c** 7-month follow-up x-rays showing distal tibial bone healing. The score calculated was 1 point (see Table 9)

In order to demonstrate the utilization of the non-union index, three cases have been selected. The first case refers to a tibial non-union in a male patient 39 years of age who sustained an open tibial fracture whilst playing football (Fig. 4). The non-union index score was 7 (Table 7). The second case refers to a 63-year-old female patient who sustained a distal 1/3 femoral fracture after a fall (Fig. 5). Here, the non-union index was 5 (Table 8). The third patient, a 44-year-old female who sustained a closed tibial fracture after a fall (Fig. 6), computed a non-union index score of 1 (Table 9).

**Table 7:** Non-union index evaluation for H.C.L. patient

LEG-NU Index	Yes	No
Site of fracture—tibia	1	
Soft tissue damage—internal degloving or open fracture	1	
Type of fracture—wedge or comminuted		0
Displacement—> 75% of shaft width	1	
Method of reduction—open	1	
Post-surgical fracture gap—> 4 mm	1	
Mechanical stability—not optimum	1	
Infection—superficial or deep	1	
Total	7

**Table 8:** Non-union index evaluation for S.M. patient

LEG-NU Index	Yes	No
Site of fracture—tibia		0
Soft tissue damage—internal degloving or open fracture	1	
Type of fracture—wedge or comminuted	1	
Displacement—> 75% of shaft width	1	
Method of reduction—open	1	
Post-surgical fracture gap—> 4 mm	1	
Mechanical stability—not optimum		0
Infection—superficial or deep		0
Total	5

**Table 9:** Non-union index evaluation for B.M. patient

LEG-NU Index	Yes	No
Site of fracture—tibia	1	
Soft tissue damage—internal degloving or open fracture		0
Type of fracture—wedge or comminuted		0
Displacement—> 75% of shaft width		0
Method of reduction—open		0
Post-surgical fracture gap—> 4 mm		0
Mechanical stability—not optimum		0
Infection—superficial or deep		0
Total	1	

## Discussion

Ten factors being post-surgical fracture gap > 4 mm, presence of infection superficial or deep, suboptimum mechanical stability, initial displacement of more than 75% of bone shaft width, fracture of the tibia rather than the femur, soft tissue damage, open method of fracture reduction, wedge or comminuted type of fracture, smoking habit, and fracture location in an area of low vascularization of the bone were evaluated. The risk factors of smoking and area of vascularization were dropped, leaving 8 risk factors that comprised an index on non-union.

The non-union index (NUI) predicts union for 0–4 risk factors and non-union for 5–8 risk factors. In practice though, early intervention might be strongly motivated when there are at least six risk factors present. All risk factors will be observable during the early outpatient clinic follow-ups following discharge, so that appropriate intervention can be undertaken three months post-operation rather than ‘wait and see’ for nine months.

Had early intervention been taken for the 47 of 200 patients included in this study, then benefit would have been seen for 46 of the 47. The other would have undergone an unnecessary operation, but may have also benefitted from increased speed of union.

The risk factors are either properties of the fracture or features of the primary surgery, apart from infection which may be modifiable through treatment.

Consequently, LEG-NUI has the great advantage of being simpler to incorporate and use in the clinical setting. Moreover, it would allow the development of a mobile APP, thus simplifying further its use during patient consultation in the clinic environment.

Effectively, a clinical decision rule or clinical decision support system (CDSS) is a system that is mainly conceived to assist physicians in taking care of patients especially helping them with clinical decision-making [38]. Although some systematic reviews have not found a real benefit in the use of CDSS [39, 40], other studies have found tangible benefits related to the use of them. For instance, Garg et al. [41] conducted a systematic review in order to assess the effectiveness of CDSS in 100 randomized and nonrandomized controlled trials. They found that the use of CDSS improved health professionals’ performance in 64% of the analysed studies. Similarly, Kawamoto et al. [42] also conducted a systematic review analysing the benefits brought by the use of CDSS in 70 randomized controlled trials. They were able to show that CDSS improved patient care and clinical practice in a high percentage (68%) of studies taken into account. Authors concluded that an important benefit of having a CDSS is that it does not provide only assessments but practical recommendations for caring of patients. In addition, the CDSS may influence decision-making since it constitutes an effective aid during the moment of a patient’s care, rather than before or after it; another important benefit of having CDSS at disposal during clinical decision-making is an improvement in the adherence to the guidelines in a long-term period [43]. Evans et al. [44] demonstrated that the use of a CDSS reduced the quantity of drugs given to the patients and their mean therapy duration time. Moreover, they found CDSS having an effective and significant impact in decreasing of costs, in terms of cost of the administered drugs and of the hospital costs, even expressed with a reduction of the mean duration of the hospital stay [44].

These findings can be applied to non-union of long-bone fractures and to the whole diagnostic and therapeutic process related to this disease. This highlights how the use of a CDSS to early diagnose and to prevent the onset of the non-union disease can be proactive to help patients to minimize the incapacity and disability-related problems and reduce health service costs.

The impact of non-union on the health-related quality of life of the patients affected by this disease is devastating. Two different recent studies [45, 46] evaluated this parameter on a big sample of patients (237 and more than 800 respectively), and they found that patients would have been disposed to trade, on average, 32% and 37% of their remaining life, in return for good health. Brinker et al. [45] found quality of life associated with tibial non-union to be comparable to the end-stage hip osteoarthritis one, whilst Schottel et al. [46] found quality of life associated with non-union as worse than the one associated with different major medical affections such as stroke, myocardial infarct, and AIDS.

Further, as suggested by Mundi et al. [47] in a commentary on Schottel et al. [46] work, the devastating economic and social consequences related to non-union are also given by the fact that most of those who develop this disease are patients at the height of their productive age. Therefore, early recognition of patients with non-union risk factors would be extremely useful for orthopaedic surgeons in order to implement close monitoring and an eventual prompt intervention to minimize risk of non-union. This kind of preventive approach would completely revolutionize the course of such a relevant and disabling disease.

It is noteworthy that such an approach has not been feasible until now not only because such an identification tool has not been available but also due to the fact that a lack of consensus in defining non-union exists [48] [49].

However, the big innovation brought by this proposed CDSS comes from the fact that it would make the preventive approach widely achievable and affordable by every orthopaedic surgeon. This does not mean that it will replace or undermine each doctor’s own clinical judgement independence, but it rather would constitute an aid, an available and ready-to-use weapon at the disposal of every orthopaedic surgeon.

Note that the assessment of the decision rule was not restricted to comparing the area under the curve, which can be insensitive [50]. Rather, evaluation considered misclassification tables, sensitivity, and specificity and balanced these against robustness and ease of use.

The risk factors considered in this study were strongly supported by the clinical literature, which was comprehensively reviewed [10, 51–54]. This study then sought to code these risk factors simply so that a simple decision rule could be derived. It is possible that more detailed consideration of each factor could contribute more information and improve the decision rule. On the other hand, the decision rule would become more complicated and therefore less applicable in clinical settings for which a simple clear guide is required.

Only records with complete details of the ten factors and the outcome of fracture healing were included in the study. These patients may have had fuller records due to concerns over their healing and therefore all at greater risk of complications such as non-union. In some respects, this potential bias strengthens our findings regarding sensitivity but may have resulted in specificity lower than would have been seen in a more representative sample (where some patients had incomplete records). Missing data though may not have been ‘missing at random’ and subsequent analysis far more complex. At the stage of deriving a suitable decision rule, as in this study, we believe that this bias is acceptable.

Two hospital sites were included for recruitment purposes. To seek more generalizability and further assessment of this decision rule, a study with a much larger sample size from multiple centres is recommended.

The fact that the CDR/LEG-NUI is based on the simple count of risk factors and is so easy to apply in the clinical environment constitutes a great strength of this study. It promises to be a powerful aid for assessing fracture fixation outcome and to support early intervention. Prediction of such an important clinical condition is envisaged to bring benefits for both commissioners and for users of health services.

## References

[1] Ekegren CL, Edwards ER, de Steiger R, Gabbe BJ (2018). Incidence, costs and predictors of non-union, delayed union and mal-union following long bone fracture. Int J Environ Res Public Health Dec.

[2] Rupp M, Biehl C, Budak M, Thormann U, Heiss C, Alt V (2018). Diaphyseal long bone nonunions - types, aetiology, economics, and treatment recommendations. Int Orthop.

[3] Zura R, Xiong Z, Einhorn T, Watson JT, Ostrum RF, Prayson MJ, Della Rocca GJ, Mehta S, McKinley T, Wang Z, Steen RG (2016). Epidemiology of fracture nonunion in 18 human bones. JAMA Surg.

[4] Busse JW, Bhandari M, Sprague S, Johnson-Masotti AP, Gafni A (2005). An economic analysis of management strategies for closed and open grade I tibial shaft fractures. Acta Orthop.

[5] Lerner RK, Esterhai JL Jr, Polomano RC, Cheatle MD, Heppenstall RB (1993) Quality of life assessment of patients with posttraumatic fracture nonunion, chronic refractory osteomyelitis, and lower-extremity amputation. Clin Orthop Relat Res (295):28–36 8403662

[6] Sprague S, Bhandari M (2002). An economic evaluation of early versus delayed operative treatment in patients with closed tibial shaft fractures. Arch Orthop Trauma Surg.

[7] Brinker MR, Browner BD, Levine AM, Jupiter JB (2003). Nonunions: evaluation and treatment. Skeletal trauma: basic science, management, and reconstruction.

[8] Hak DJ, Fitzpatrick D, Bishop JA, Marsh JL, Tilp S, Schnettler R, Simpson H, Alt V (2014). Delayed union and nonunions: epidemiology, clinical issues, and financial aspects. Injury.

[9] Pountos I, Georgouli T, Pneumaticos S, Giannoudis PV (2013). Fracture non-union: can biomarkers predict outcome?. Injury.

[10] Santolini E, West R, Giannoudis PV (2015). Risk factors for long bone fracture non-union: a stratification approach based on the level of the existing scientific evidence. Injury.

[11] Peduzzi P, Concato J, Kemper E, Holford TR, Feinstein AR (1996). A simulation study of the number of events per variable in logistic regression analysis. J Clin Epidemiol.

[12] Macnab I, De Haas WG (1974). The role of periosteal blood supply in the healing of fractures of the tibia. Clin Orthop Relat Res.

[13] Santolini E, Goumenos SD, Giannoudi M, Sanguineti F, Stella M, Giannoudis PV (2014). Femoral and tibial blood supply: a trigger for non-union?. Injury.

[14] National Healthcare Safety Network CDC (2017) Surgical site infection (SSI) event. Procedure-associated Module. http://www.cdc.gov/nhsn/pdfs/pscmanual/9pscssicurrent.pdf. Accessed 5 Jan 2019

[15] Zou J, Zhang W, Zhang CQ (2013). Comparison of minimally invasive percutaneous plate osteosynthesis with open reduction and internal fixation for treatment of extra-articular distal tibia fractures. Injury.

[16] Gustilo RB, Anderson JT (1976). Prevention of infection in the treatment of one thousand and twenty-five open fractures of long bones: retrospective and prospective analyses. J Bone Joint Surg Am.

[17] Giannoudis PV, Einhorn TA, Marsh D (2007). Fracture healing: the diamond concept. Injury.

[18] Ahmad M, Nanda R, Bajwa AS, Candal-Couto J, Green S, Hui AC (2007). Biomechanical testing of the locking compression plate: when does the distance between bone and implant significantly reduce construct stability?. Injury.

[19] Bong MR, Kummer FJ, Koval KJ, Egol KA (2007). Intramedullary nailing of the lower extremity: biomechanics and biology. J Am Acad Orthop Surg.

[20] Fleming B, Paley D, Kristiansen T, Pope M (1989) A biomechanical analysis of the Ilizarov external fixator. Clin Orthop Relat Res (241):95–105 2924484

[21] Fragomen AT, Rozbruch SR (2007). The mechanics of external fixation. HSS J.

[22] Freeman AL, Tornetta P, Schmidt A, Bechtold J, Ricci W, Fleming M (2010). How much do locked screws add to the fixation of “hybrid” plate constructs in osteoporotic bone?. J Orthop Trauma.

[23] Gautier E, Sommer C (2003). Guidelines for the clinical application of the LCP. Injury.

[24] Penzkofer R, Maier M, Nolte A, von Oldenburg G, Puschel K, Buhren V, Augat P (2009). Influence of intramedullary nail diameter and locking mode on the stability of tibial shaft fracture fixation. Arch Orthop Trauma Surg.

[25] Podolsky A, Chao EY (1993) Mechanical performance of Ilizarov circular external fixators in comparison with other external fixators. Clin Orthop Relat Res (293):61–70 8339510

[26] Rüedi T, Murphy W (2001). AO principles of fracture management.

[27] Stoffel K, Dieter U, Stachowiak G, Gachter A, Kuster MS (2003). Biomechanical testing of the LCP--how can stability in locked internal fixators be controlled?. Injury.

[28] Xia L, Zhou J, Zhang Y, Mei G, Jin D (2014). A meta-analysis of reamed versus unreamed intramedullary nailing for the treatment of closed tibial fractures. Orthopedics.

[29] Salem KH (2012). Critical analysis of tibial fracture healing following unreamed nailing. Int Orthop.

[30] Castillo RC, Bosse MJ, MacKenzie EJ, Patterson BM (2005). Impact of smoking on fracture healing and risk of complications in limb-threatening open tibia fractures. J Orthop Trauma.

[31] Schmitz MA, Finnegan M, Natarajan R, Champine J (1999). Effect of smoking on tibial shaft fracture healing. Clin Orthop Relat Res.

[32] Gustilo RB, Mendoza RM, Williams DN (1984). Problems in the management of type III (severe) open fractures: a new classification of type III open fractures. J Trauma.

[33] Kellam J, Audigé L, Rüedi TP, Buckley RE, Moran CG (2007). Fracture classification. AO principles of fracture management.

[34] Clegg A, Bates C, Young J, Ryan R, Nichols L, Ann Teale E, Mohammed MA, Parry J, Marshall T (2016). Development and validation of an electronic frailty index using routine primary care electronic health record data. Age Ageing.

[35] Dawes RM (1979). The robust beauty of improper linear models in decision making. Am Psychol.

[36] Team RC (2016). R: a language and environment for statistical computing.

[37] Sólymos Péter, Lele Subhash R. (2015). Revisiting resource selection probability functions and single-visit methods: clarification and extensions. Methods in Ecology and Evolution.

[38] Berner ES (2007) Clinical decision support systems: theory and practice. Springer Science & Business Media, pp 3–22

[39] Black AD, Car J, Pagliari C, Anandan C, Cresswell K, Bokun T, McKinstry B, Procter R, Majeed A, Sheikh A (2011). The impact of eHealth on the quality and safety of health care: a systematic overview. PLoS Med.

[40] Moja L, Kwag KH, Lytras T, Bertizzolo L, Brandt L, Pecoraro V, Rigon G, Vaona A, Ruggiero F, Mangia M, Iorio A, Kunnamo I, Bonovas S (2014). Effectiveness of computerized decision support systems linked to electronic health records: a systematic review and meta-analysis. Am J Public Health.

[41] Garg AX, Adhikari NK, McDonald H, Rosas-Arellano MP, Devereaux PJ, Beyene J, Sam J, Haynes RB (2005). Effects of computerized clinical decision support systems on practitioner performance and patient outcomes: a systematic review. Jama.

[42] Kawamoto K, Houlihan CA, Balas EA, Lobach DF (2005). Improving clinical practice using clinical decision support systems: a systematic review of trials to identify features critical to success. BMJ (Clin Res Ed).

[43] Nachtigall I, Tafelski S, Deja M, Halle E, Grebe MC, Tamarkin A, Rothbart A, Uhrig A, Meyer E, Musial-Bright L, Wernecke KD, Spies C (2014). Long-term effect of computer-assisted decision support for antibiotic treatment in critically ill patients: a prospective ‘before/after’ cohort study. BMJ Open.

[44] Evans RS, Pestotnik SL, Classen DC, Clemmer TP, Weaver LK, Orme JF, Lloyd JF, Burke JP (1998). A computer-assisted management program for antibiotics and other antiinfective agents. N Engl J Med.

[45] Brinker MR, Hanus BD, Sen M, O'Connor DP (2013). The devastating effects of tibial nonunion on health-related quality of life. J Bone Joint Surg Am.

[46] Schottel PC, O'Connor DP, Brinker MR (2015). Time trade-off as a measure of health-related quality of life: long bone nonunions have a devastating impact. J Bone Joint Surg Am.

[47] Mundi R, Bhandari M (2015). Devastating impact of fracture nonunions: the need for timely identification and intervention for high-risk patients: commentary on an article by Patrick C. Schottel, MD, et al.: “time trade-off as a measure of health-related quality of life: long bone nonunions have a devastating impact”. J Bone Joint Surg Am.

[48] Corrales L, Morshed S, Bhandari M, Tr M (2008). Variability in the assessment of fracture-healing in orthopaedic trauma studies. J Bone Joint Surg Am.

[49] Bhandari M, Guyatt G, Swiontkowski M, Pr T, Sprague S, Schemitsch E (2002). A lack of consensus in the assessment of fracture healing among orthopaedic surgeons. J Orthop Trauma.

[50] Cook NR (2007). Use and misuse of the receiver operating characteristic curve in risk prediction. Circulation.

[51] Ryu Seung Min, Choi Chang Hyun, Yang Han Seok, Park Wook Tae, Shon Oog Jin, Park Sam-Guk (2018). Causes and treatment outcomes of revision surgery after open reduction and internal fixation of tibial plateau fractures. International Orthopaedics.

[52] Hosny GA, Ahmed AA, Hussein MA (2018). Clinical outcomes with the corticotomy-first technique associated with the Ilizarov method for the management of the septic long bones non-union. Int Orthop.

[53] Allende C, Vanoli F, Gentile L, Gutierrez N (2018). Minimally invasive plate osteosynthesis in humerus nonunion after intramedullary nailing. Int Orthop.

[54] Koso RE, Terhoeve C, Steen RG, Zura R (2018). Healing, nonunion, and re-operation after internal fixation of diaphyseal and distal femoral fractures: a systematic review and meta-analysis. Int Orthop Nov.

